# Bayesian analysis of one‐inflated models for elusive population size estimation

**DOI:** 10.1002/bimj.202100187

**Published:** 2022-03-25

**Authors:** Tiziana Tuoto, Davide Di Cecco, Andrea Tancredi

**Affiliations:** ^1^ Istat ‐ Istituto nazionale di statistica Rome Italy; ^2^ Department of Methods and Models for Economics Territory and Finance Sapienza University of Rome Rome Italy

**Keywords:** Bayesian model selection, capture–recapture, illegal populations, zero‐truncated one‐inflated count data models

## Abstract

The identification and treatment of “one‐inflation” in estimating the size of an elusive population has received increasing attention in capture–recapture literature in recent years. The phenomenon occurs when the number of units captured exactly once clearly exceeds the expectation under a baseline count distribution. Ignoring one‐inflation has serious consequences for estimation of the population size, which can be drastically overestimated. In this paper we propose a Bayesian approach for Poisson, geometric, and negative binomial one‐inflated count distributions. Posterior inference for population size will be obtained applying a Gibbs sampler approach. We also provide a Bayesian approach to model selection. We illustrate the proposed methodology with simulated and real data and propose a new application in official statistics to estimate the number of people implicated in the exploitation of prostitution in Italy.

## 1 INTRODUCTION

A popular methodology to estimate the size of an elusive population is the capture–recapture method, originally used to estimate animal abundance. When the captures/observations are continuously collected over a fixed interval of time, and time is considered uninfluential, the total number of captures for each unit is the sufficient statistic. Here we focus on this setting, usually called “repeated counting data” (Böhning & Schön, 2005). To estimate the population size, the observation/capturing counting process must first be modeled.

In Farcomeni and Scacciatelli (2013), “one–inflation” is explicitly mentioned for criminal populations as a (simple) particular case in a broader class of behavioral effects. In more recent years, a series of papers—see, for example, Godwin and Böhning (2017), Godwin (2017), Godwin (2019), Böhning et al. (2018), and Böhning and Friedl (2021)—have been devoted specifically to the phenomenon in repeated counting data.

One‐inflation consists in an excess of “ones” in the observed data, that is, more units than expected are captured exactly once. The excess of “ones” is usually evaluated with respect to a chosen family of counting distributions: Godwin and Böhning (2017) considered one‐inflation with respect to a “base” Poisson model, while Böhning and Friedl (2021) analyzed the inflation in the geometric case. One‐inflated negative binomial was introduced in Godwin (2017), and the finite mixture of one‐inflated Poissons (OIPs) in Godwin (2019).

One‐inflation can occur for different reasons; for instance, when some units of the population can no longer be captured after the first capture. Such may be the case of some wild animal populations. In fact, animals experiencing a capture may find it so unpleasant that some develop the will and ability to avoid subsequent captures. Much the same mechanism may also occur in human populations, particularly when the first capture is a matter of law enforcement, involves imprisonment, or reveals an undesirable characteristic/behavior. See Godwin and Böhning (2017) for ample discussion of the justifications and conditions for one‐inflation in capture–recapture, also including an interpretation of one‐inflation as limiting case of the so‐called “trap shy” behavioral model; see, for example, p. 37 of McCrea and Morgan (2014) or p. 119 of Borchers et al. (2002). One‐inflation deserves specific attention due to its effect on population size estimators. In fact, when not taken into account, one‐inflation causes overestimation of the total population size. This also applies to the well‐known lower bound Chao estimator, as discussed in Chiu and Chao (2016) and Böhning et al. (2018).

In this paper we propose a Bayesian approach for counting data models with one‐inflation. The properties of our models are analyzed with both simulation studies and real data applications. In particular, we apply our models to real data to estimate the size of some illegal populations active in Italy in 2014 and some real data available from the literature on capture–recapture, where the issue of one‐inflation has been recognized.

The paper is organized as follows: In Section 2 we introduce the notation for repeated counting data and broadly illustrate Bayesian inference for population size with this kind of data. We describe the general model for one‐inflated count data under an unspecified counting distribution and outline a Gibbs sampler algorithm to handle the one‐inflated models. We also introduce a formal Bayesian procedure for model comparison in the presence of one‐inflated models. Section 3 specifies the results under the Poisson and geometric assumptions, corroborating our proposal with a simulation study. In Section 4 we introduce the negative binomial distribution and its one‐inflated counterpart discussing the boundary problem via a simulation study. In Section 5 we illustrate some applications to real cases: First we show the results of our inference on data on prostitution exploitation in Italy in 2014; moreover, we apply our models to some popular data sets in capture–recapture literature. Section 6 concludes the paper with some remarks and discussion of open issues for further investigation.

## 2 BAYESIAN INFERENCE FOR POPULATION SIZE

According to the standard formulation, consider a closed population (no births, deaths, or migration) of size N. For each unit in the population, let Y be a random variable taking value j=0,1,2,⋯ if the individual is observed/captured j times. We only observe the n individuals, n≤N, which are captured at least once. Let $y=(y_{1},\text{…},y_{n})$ be the vector of the individual number of captures. Note that y will denote the result of the capture–recapture experiment, which comprises both the number n of captured individuals and the number of captures for each observed individual.

Let $n_{j}$ denote the number of individuals observed j times, that is, $n_{j}$ is the frequency of count j in sample y. Our interest is to estimate the number of uncaptured units $n_{0}$, and, consequently, the total population size $N=n+n_{0}$, on the basis of some model for the observed $n_{j}$.

Bayesian inference for the population size N can be obtained with standard Markov chain Monte Carlo (MCMC) algorithms. In fact, let f(y|θ)=P(Y=y|θ) for y=0,1…, be the probability distribution function for Y. The generic expression for the likelihood f(y|θ,N) is

(1) f(y|θ,N)=Nnf(0|θ)N−n∏i=1nf(yi|θ).

Assuming independent priors for θ and N, that is, p(θ,N)=p(θ)p(N), the posterior distribution p(θ,N|y) can easily be drawn by, for example, updating the conditional distributions

$$p\left(\theta |N,y\right)\propto f{\left(0|\theta \right)}^{N-n}\prod_{i=1}^{n}f\left(y_{i}|\theta \right)\;p\left(\theta \right)$$

and

p(N|θ,y)∝Nnf(0|θ)N−np(N).

We can generate from those posteriors via Gibbs or Metropolis–Hastings steps, according to the parametric family for Y and the prior for N.

In the Bayesian literature, common choices for the (default or noninformative) prior over N are:

(1) $p\left(N\right)\propto N^{l}$ for l∈{−2,−1,−1/2,0} possibly truncating the prior to an opportune upper bound; l=−1 corresponds to the Jeffreys' prior which is improper;
(2) Rissanen's prior (Rissanen, 1983), which is always proper and is given by $p\left(N\right)\propto 2^{-\log^{*}\left(N\right)}$, where $\log^{*}\left(N\right)$ is the sum of the positive terms in the sequence $\left\{\log_{2}\left(N\right),\log_{2}\left(\log_{2}\left(N\right)\right),\text{…}\right\}$.

See Tardella (2002), Wang et al. (2007), and Xu et al. (2014) for extensive simulation studies.

Note the following:

(1) by assuming p(N)∝1/N, the full conditional distribution of $n_{0}=N-n$ is negative binomial with size parameter n and probability f(0|θ) whatever the model for Y may be;
(2) the full conditional of θ corresponds to its posterior distribution when the zero counts are also known.

For example, when Y is Poisson(λ) and a priori we take the conjugate prior for λ, which is Gamma$\left(\alpha_{\lambda },\beta_{\lambda }\right)$, the latter step consists solely in the generation of a Gamma distribution with parameters given by $\alpha_{\lambda }+s$ and $\beta_{\lambda }+n+n_{0}$, where s is the sum of the observed captures. Similarly, when Y is geometric(p) and a priori we take the conjugate prior for p, which is Beta$\left(\alpha_{p},\beta_{p}\right)$, this step consists in the generation of a Beta distribution with parameters $\alpha_{p}+n+n_{0}$ and $\beta_{p}+s$.

### 2.1 One‐inflated models

We assume that in our population a specific behavioral mechanism is at work, by virtue of which an individual that would otherwise face multiple captures now has a positive probability ω of being captured just once.

Let Y denote the observed number of captures for a unit, and $Y^{*}$ the latent value we would observe without the behavioral mechanism. The two variables are linked by means of the following infinite transition matrix:

$$\left(\begin{array}{cccccc} 1 & 0 & 0 & 0 & 0 & \cdots \\ 0 & 1 & 0 & 0 & 0 & \cdots \\ 0 & \omega & 1-\omega & 0 & 0 & \cdots \\ 0 & \omega & 0 & 1-\omega & 0 & \cdots \\ 0 & \omega & 0 & 0 & \ddots & \;\\ \vdots & \vdots & \vdots & \vdots & & \; \end{array}\right),$$

where the (k,j)th element represents the conditional probability $P(Y=j-1\;|\;\omega ,Y^{*}=k-1)$. When k>1 these conditional probabilities can be written as

$$P\left(Y=j\;|\;\omega ,Y^{*}=k\right)=\omega^{\left(1-\delta_{k}\left(j\right)\right)}{\left(1-\omega \right)}^{\delta_{k}\left(j\right)}\;j=1,k,$$

where $\delta_{k}\left(j\right)$ is Kronecker delta.

Let $f\left(k|\theta \right)=P\left(Y^{*}=k\;|\;\theta \right)$ be the probability distribution, depending on a given parameter, θ, of the number of captures without the behavioral effect, and let F(θ) denote the associated c.d.f. Then, the resulting distribution for Y is the one‐inflated model defined as follows:

$$P\left(Y=j\;|\;\theta ,\omega \right)=\left\{\begin{array}{cc} f(0|\theta ) & \text{if}\;j=0;\\ \left(1-\omega )f(1|\theta )+\omega (1-f(0|\theta )\right) & \text{if}\;j=1;\\ \left(1-\omega )f(j|\theta \right) & \text{if}\;j>1. \end{array}\right.$$

The conditional distribution of $Y^{*}$ when Y=j is concentrated on j when j≠1, while, when j=1, we have:

(2) $$P\left(Y^{*}=k\;|\;Y=1,\theta ,\omega \right)=\left\{\begin{array}{cc} 0 & \text{if}\;k=0;\\ \frac{f(1|\theta )}{f(1|\theta )+\omega (1-F(1|\theta ))} & \text{if}\;k=1;\\ \frac{\omega f(k|\theta )}{f(1|\theta )+\omega (1-F(1|\theta ))} & \text{if}\;k>1. \end{array}\right.$$

### 2.2 Gibbs sampler for one‐inflated models

Bayesian inference for one‐inflated models can be obtained by simulating the posterior distribution of $\theta ,\omega ,N,y_{1}^{*},\text{…},y_{n}^{*}$ given the observed data y, where $y_{1}^{*},\text{…},y_{n}^{*}$ indicate the unknown captures that the n observed units would have faced without the behavioral mechanism. Let us assume that the parameters θ,ω, and N are a priori independent and let p(θ,ω,N)=p(ω)p(θ)p(N) denote the prior distribution. The general expression for the posterior distribution of one‐inflated models augmented with the vector $y^{*}=\left(y_{1}^{*},\text{…},y_{n}^{*}\right)$ is

p(θ,ω,N,y∗|y)∝p(y|θ,ω,N,y∗)p(y∗,θ,ω,N)∝∏i=1nP(Yi=yi|yi∗,ω)p(y∗|N,θ)p(θ)p(ω)p(N)∝Nnf(0|θ)N−n∏i=1nP(Yi=yi|yi∗,ω)f(yi∗|θ)p(θ)p(ω)p(N).

To describe our approach to simulate the posterior distribution of one‐inflated models, we introduce an additional latent binary variable $Z_{i}$ indicating the presence/absence of the behavioral mechanism, which causes the one‐inflation in unit i, that is, $Z_{i}$ is the indicator function of the event $\left\{Y_{i}\neq Y_{i}^{*}\right\}$. We then have that:

$$P(Z_{i}=1\;|\;Y_{i}\neq 1)=0,$$

and, from (2), we have

$$P\left(Z_{i}=1\;|\;Y_{i}=1\right)=\frac{\omega (1-F(1|\theta ))}{f(1|\theta )+\omega (1-F(1|\theta ))}.$$

Then, since $Z_{i}=1$ implies $Y_{i}^{*}>1$, we have

(3) $$P\left(Y_{i}^{*}=k\;|\;Z_{i}=1\right)=\left\{\begin{array}{cc} \frac{f(k\;|\;\theta )}{1-F(1\;|\;\theta )} & \text{if}\;k>1;\\ 0 & \text{otherwise}. \end{array}\right.$$

We can now outline a Gibbs sampler looping over the full conditionals of $Y^{*}$ and ω, N, and θ. The updating of θ will depend on the model assumption for $Y^{*}$ and may require a Metropolis‐within‐Gibbs step, whereas the updating of $Y^{*}$, ω, and N can always be performed with the following exact Gibbs steps:

(1) The simulation of the full conditional of $Y_{1}^{*},\text{…},Y_{n}^{*}$ can be obtained in two steps, by first updating $Z_{1},\text{…},Z_{n}$. In fact, let $n_{z}=\sum_{i=1}^{n}Z_{i}$ be the number of units affected by one–inflation; then, conditional on the current value of ω and θ, we can generate a value for $n_{z}$ from
$$Binom\left(n_{1}\;,\;\frac{\omega (1-F(1|\theta ))}{f(1|\theta )+\omega (1-F(1|\theta ))}\right).$$
  Then, for each of the $n_{z}$ units, we can generate a value of $Y^{*}$ by simply simulating a number of captures from the truncated count distribution (3).
(2) Consider the prior
$$\omega ∼Beta(\alpha_{\omega },\beta_{\omega }),$$
  and let $n_{z,k}$ be the number of units among the $n_{z}$ for which $Y^{*}=k$, such that $\sum_{k}n_{z,k}=n_{z}$. We can then write the full conditional of ω, p(ω|−) as:
$$p\left(\omega \;|\;-\right)\propto \omega^{\alpha_{\omega }-1}{\left(1-\omega \right)}^{\beta_{\omega }-1}\;\prod_{k>1}{\left[\omega f(k\;|\;\theta )\right]}^{n_{z,k}}\cdot {\left[(1-\omega )f(k\;|\;\theta )\right]}^{n_{k}}.$$
  That is, we can directly draw ω from
$$Beta\left(\alpha_{\omega }+n_{z}\;,\;\beta_{\omega }+\sum_{k>1}n_{k}\right).$$
(3) The full conditional distribution of N is given by
p(N|−)∝Nnf(0|θ)N−np(N)
  and, by assuming the improper prior p(N)∝1/N we can directly draw $n_{0}$ from the following negative binomial
N−1n−1f(0|θ)N−n(1−f(0|θ))n.
  If we adopt a different prior over N, we have to implement a Metropolis step.

Finally, as we have seen, the updating of θ will depend on the model assumption for $Y^{*}$. The general expression for the full conditional of θ is:

$$p\left(\theta \;|\;-\right)\propto f{\left(0|\theta \right)}^{N-n}\prod_{i=1}^{n}f\left(Y_{i}^{*}|\theta \right)p\left(\theta \right).$$

### 2.3 Model selection

To test the one‐inflation assumption with respect to a specific base count distribution we can adopt a fully Bayesian approach. Let $M_{1}$ be the noninflated model and $M_{2}$ the one‐inflated counterpart (indicated by the OI suffix, hereafter). Model comparison can be performed by calculating the posterior model probabilities

$$P\left(M_{i}\;|\;y\right)=\frac{p\left(M_{i}\right)p\left(y|M_{i}\right)}{p\left(M_{1}\right)p\left(y|M_{1}\right)+p\left(M_{2}\right)p\left(y|M_{2}\right)},$$

where $p(y|M_{i})$ is the marginal likelihood that, for the models considered in this paper, can be generally written as

$$p\left(y|M_{i}\right)=\int \sum_{N=n}^{\infty }f\left(y\;|\;\theta_{i},N,M_{i}\right)p\left(\theta_{i},N\;|\;M_{i}\right)\;d\theta_{i},$$

with $\theta_{1}$ and $\theta_{2}$ denoting, respectively, the parameters of the baseline and the OI counterpart models. For instance, for Poisson model we have $\theta_{1}=\lambda$ and $\theta_{2}=\left(\lambda ,\omega \right)$, for the geometric case we have $\theta_{1}=p$ and $\theta_{2}=\left(p,\omega \right)$. In the case of two models we can directly use the Bayes factor (BF) in favor of the OI

$$BF=\frac{P(M_{2}\;|\;y)}{P(M_{1}\;|\;y)}=\frac{P(M_{2})}{P(M_{1})}\frac{p(y|M_{2})}{p(y|M_{1})}.$$

Note that we can also extend the comparison setting by simultaneously considering more than two models. For example, in the next section we compare the Poisson and the geometric model together with the corresponding OI counterparts for a total of four models. Assuming equal prior probabilities $P(M_{i})$ for i=1,…,k, the posterior model probabilities are proportional to the marginal likelihoods, that is, $P\left(M_{i}\;|\;y\right)\propto p\left(y|M_{i}\right)$ for i=1,…,k. Note. moreover, that assuming the noninformative prior p(N)=c/N would produce marginal likelihoods depending on the constant c. However, in our case, the parameter N has the same meaning across all the models under comparison, hence the use of the same improper prior p(N)=c/N is justified and the constant c cancels out in the evaluation of the posterior model probabilities, see Kass and Raftery (1995).

Analytical evaluation of the marginal likelihoods $p(y|M_{i})$ is not possible. However, we have that (see the Appendix)

(4) $$p\left(y|M_{i}\right)=c\int \sum_{N=n}^{\infty }f\left(y|\theta_{i},N,M_{i}\right)\frac{1}{N}p\left(\theta_{i}\right)\;d\theta_{i}=\frac{c}{n}\int \prod_{i=1}^{n}\frac{f(y_{i}|\theta_{i})}{1-f(0|\theta_{i})}p\left(\theta_{i}\right)d\theta_{i}.$$

Hence, the posterior model probabilities will depend solely on fitting the truncated distribution of Y to the observed captures.

To evaluate the marginal likelihood of each model numerically, we use the Chib's approximation introduced in Chib (1995), which can easily be obtained as a by‐product of the general Gibbs algorithm illustrated in the previous section. The details of the Chib approximation for all the models considered throughout this paper are given in the Appendix.

Finally, it is worth noting that, in the context of capture–recapture, model averaging appears to be a suitable alternative to model selection. In fact, the quantity of interest N has the same meaning across different models and we can easily obtain an estimate $\bar{N}$ of N averaged over the eligible alternatives via the following formula:

$$\bar{N}=E\left[N\;|\;y\right]=\sum_{i}{\hat{N}}_{M_{i}}\;P\left(M_{i}\;|\;y\right),$$

where ${\hat{N}}_{M_{i}}$ is the posterior mean of N obtained under model $M_{i}$. However, since the estimates of N under the base model and under its one‐inflated counterpart may show very considerable differences, definite choice between the two could be a sensible approach in this case.

## 3 ONE‐INFLATED POISSON AND GEOMETRIC DISTRIBUTIONS

If we assume that our count data $Y^{*}$ follows a Poisson distribution, that is, f(θ) represents a Poisson density with parameter λ, the model proposed for the observed Y in previous Section 2.1 is an OIP and corresponds to the model presented in Godwin and Böhning (2017).

The estimating procedure is based on the Gibbs sampler described in Section 2.1, where, in order to complete the analysis framework, we assume a Gamma($\alpha_{\lambda },\beta_{\lambda }$) prior for λ, $\alpha_{\lambda }$, and $\beta_{\lambda }$ being shape and rate parameters. Let $n_{k}^{*}$ be the total number of units captured k times after updating $n_{0}$, $n_{z}$, and $Y^{*}$, that is,

$$n_{k}^{*}=\left\{\begin{array}{cc} n_{0} & \text{for}\;k=0;\\ n_{1}-n_{z} & \text{for}\;k=1;\\ n_{k}+n_{z,k} & \text{if}\;k>1; \end{array}\right.$$

and let $\left\{n^{*}\right\}$ denote the set of all values $n_{k}^{*}$ for k=0,1,… We can then generate the updated value for λ from its full conditional

$$Gamma\left(\alpha_{\lambda }+\sum_{k>0}k\;n_{k}^{*}\;,\;\beta_{\lambda }+N\right).$$

If we adopt a geometric distribution for $Y^{*}$, parameterized as

$$P\left(Y^{*}=k\;|\;p\right)={\left(1-p\right)}^{k}p,$$

the resulting model for Y is called one–inflated geometric (OIG). To finalize the Bayesian analysis, we adopt a $Beta(\alpha_{p},\beta_{p})$ conjugate prior for p, and its posterior conditional on the current values of $n_{0}$, $n_{z}$, and $Y^{*}$ would be equal to:

$$Beta\left(\alpha_{p}+N\;,\;\beta_{p}+\sum_{k>0}k\;n_{k}^{*}\right).$$

### 3.1 A simulation study

In this section we present a twofold simulation study; on one hand, we aim to validate our proposal for inference on the population size in the presence of one‐inflation, while on the other hand the results of the simulation study illustrate the model selection among the four models presented in the previous section, namely, Poisson (which we refer to as model Poi), Geometric (Geo), OIP, and OIG. Specifically, we set up three main scenarios: In the first we generate from the base distributions without one‐inflation; in the second scenario, we generate from one‐inflated distributions with a low/moderate inflation rate (ω=0.2), while in the third we consider a substantial inflation rate (ω=0.5). We repeat each scenario with two different values of the parameter (λ or p) and with two different values of N (500 and 1000). We set the parameters using values similar to those from the real cases analyzed in Section 5. The scenarios and the values of the different parameters are summarized in Table 1.

**TABLE 1:** Simulation scenarios with data‐generating models, parameter values, and expected sample size E[n] (the expected values of n are common to all three scenarios)

Scenario I	Scenario II	Scenario III	Distribution		
No inflation	Low inflation, ω=0.2	Substantial inflation, ω=0.5	N	Parameter	E[n]
Poi	OIP	OIP	500	λ=1	316
				λ=2	432
			1000	λ=1	632
				λ=2	865
Geo	OIG	OIG	500	p=0.4	300
				p=0.6	200
			1000	p=0.4	600
				p=0.6	400

For each combination of parameters in each scenario we simulate 100 data sets of N units from the respective generating model and remove the 0 counts from the sample. To simulate from the one‐inflated models in Scenarios II and III, we generate from the corresponding base model and then change each generated value greater than 1 to a 1 with probability ω. All the experiments were conducted in R and the code is available as Supporting Information on the journal's web page.

First, we set out to evaluate the sensitiveness of the estimates of the unobserved population size $n_{0}$ under mispecification of the model. For each simulated data set, we consider the estimates of $n_{0}$, given by the posterior mean, under all four models, and compute relative bias calculated as the relative difference between the true value and the posterior mean of the parameter. Table 2 shows the average percentage relative bias over the 100 replicates.

**TABLE 2:** Relative bias (%) of the unobserved units estimates, $n_{0}$

Generating model			N=500				N=1000			
Model	Parameter	Inflation	Poi	Geo	OIP	OIG	Poi	Geo	OIP	OIG
Poi	1	None	1.67	198	−12	189	0.37	196	−9	190
Poi	2	None	1.28	391	−5.49	389	0.88	390	−4.12	388
Geo	0.4	None	−82	−0.80	−91	−5.48	−82	−1.13	−91	−4.33
Geo	0.6	None	−68	0.27	−80	−9.34	−68	0.73	−82	−6.84
OIP	1	0.2	52	514	3.41	501	52	514	2.32	507
OIP	2	0.2	37	273	0.71	246	37	272	0.38	254
OIP	1	0.5	147	497	14	339	146	496	6.04	146
OIP	2	0.5	218	883	5.38	619	219	886	3.54	614
OIG	0.4	0.2	−72	25	−91	0.92	−73	23	−91	−0.03
OIG	0.6	0.2	−55	26	−79	1.50	−56	26	−81	1.21
OIG	0.4	0.5	−39	100	−91	1.72	−39	100	−91	2.07
OIG	0.6	0.5	−16	108	−76	15	−18	104	−79	7.74

The results set out in Table 2 confirm that the estimates of $n_{0}$ we obtain with a one‐inflated model are always lower than those obtained with the corresponding base model. In fact, ignoring one‐inflation when present leads to severe and systematic overestimate of $n_{0}$. On the other hand, admitting one‐inflation when it is not present is not such a serious error and, on average, we moderately underestimate $n_{0}$. Choosing the wrong model (Poisson instead of geometric, inflated or not) can have disastrous consequences. In particular, if data come from Poi or OIP models, a Geo or OIG model would drastically overestimate $n_{0}$. If data are generated from a Geo or OIG model, choosing a Poi or OIP model implies an equivalent underestimate of $n_{0}$. Note that, the two cases having the highest relative bias under the correct models can be justified by the observed number of captures. In particular, when the generating model is OIP with λ=1 and ω=0.5, the expected number of captured units is low (E[n]=316 when N=500), and most of them are captured exactly once ($E[n_{1}]=250$). The same happens in the case of OIG with p=0.6 and ω=0.5 where E[n]=200 and $E[n_{1}]=160$. However, even in these worst cases, the relative bias decreases, as expected, when the sample size increases.

Here we will not present the results concerning the relative root mean squared error and the relative mean absolute error, which in any case, confirm the results presented on the relative bias.

These results are also confirmed on analyzing the coverage of the posterior credible intervals, not reported here for brevity but computed by the R code available in the Supporting Information on the journal's web page. The posterior credible intervals of the one‐inflated model almost always contain the true values when we generate from the corresponding baseline distribution. On the other hand, when we generate from a one‐inflated model, the credible intervals of the baseline model barely cover the true values. The credible intervals deriving from the Poisson models (regardless of one‐inflation) seldom cover the true value generated by the geometric distribution, and vice versa. The only exception is the case in which we generate from OIG (p=0.6, ω=0.5) and estimate with a Poisson distribution (see the bottom row in Table 2), in which case the baseline Poisson credible intervals cover the true value nearly 50% of the times.

**FIGURE 1:**
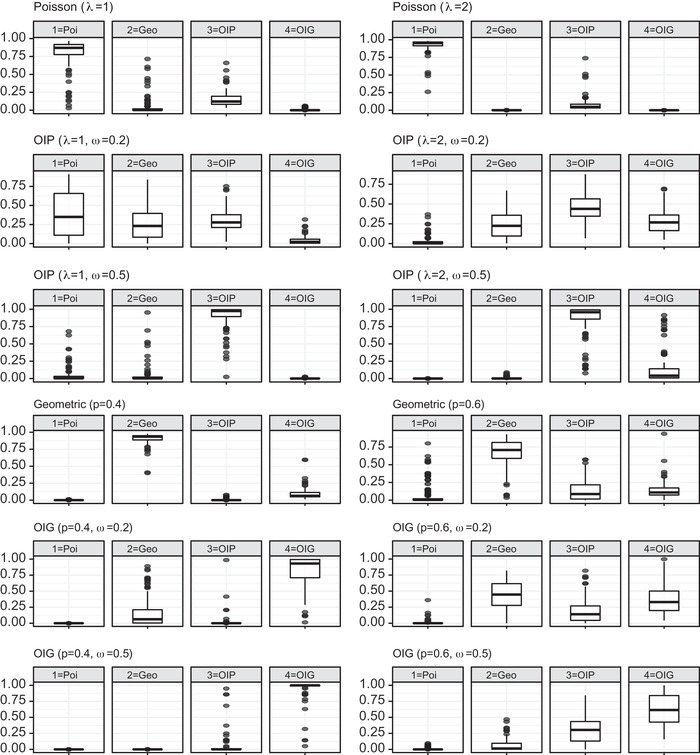
Box‐plot of posterior model probabilities when N=500; the data‐generating model is indicated above each panel

**FIGURE 2:**
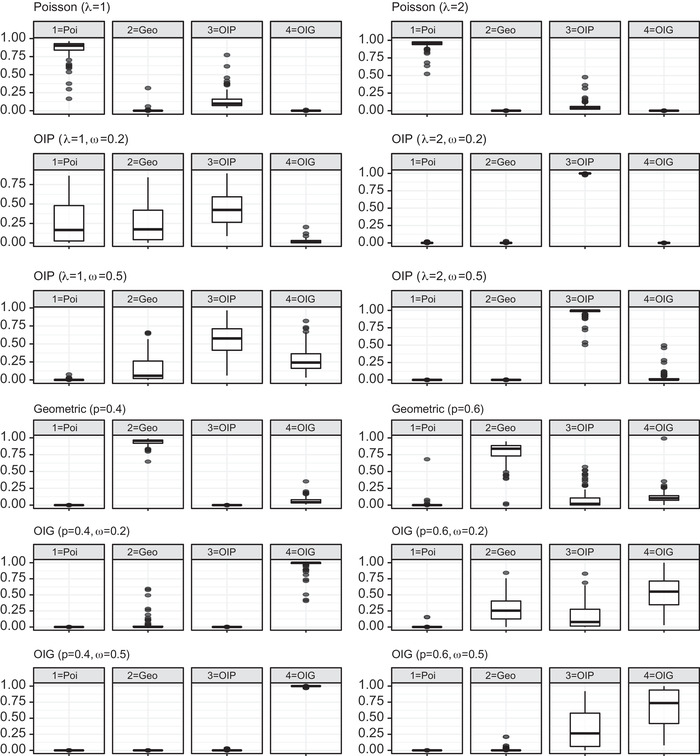
Box‐plot of posterior model probabilities when N=1000; the data‐generating model is indicated above each panel

Next, to assess the model selection criterion detailed in the previous section, Figures 1 and 2 show the posterior probabilities of our four competing models calculated with Chib's approximation. Figure 1 summarizes the results in all the scenarios when N=500, while Figure 2 refers to the case N=1000.

It is evident that, as the number of observed units n increases, the effectiveness of the posterior model probabilities in identifying the correct generating model is reinforced. Note that n depends both on N and on the parameters λ and p. It is also evident that a higher inflation rate will be more easily identified correctly. In fact, when N=1000, we would select the true data‐generating model in almost all simulations in Scenarios I and III, and in most cases in Scenario II. For the sake of brevity, here we do not present the results when N=2000 or higher, since in all scenarios and parameter combinations the posterior model probability of the generating model is close to one.

When N=500, we would still identify the correct generating model in the majority of cases, but we can observe some critical situations. In particular, when the generating model is OIP with λ=1 and ω=0.2, and when we generate from the OIG with p=0.6 and ω=0.2, the correct model and its base counterpart are almost equally preferable. In the former case we have n=316 and $n_{1}=183$ on average, that is, most of the units are captured once. Consequently, the posterior probabilities are very similar due to such a slight alteration in singleton counts from the basic Poisson distribution. Much the same happens in the latter case, with an even lower number of observations (on average n=200).

For a simulation study using frequentist criteria for model selection (Akaike information criterion [AIC] and Bayesian information criterion [BIC]) see Böhning and Ogden (2021).

In conclusion, as expected, the one‐inflation models encompass the baseline models and, when one‐inflation is not present, the slight underestimation of N decreases as n increases. Clearly, the choice of the distribution is a crucial aspect, and the Bayesian approach gives us a powerful tool to deal with model selection.

## 4 ONE‐INFLATED NEGATIVE BINOMIAL

In this section we describe how to perform Bayesian estimation of the population size in the presence of one‐inflation when the base distribution is the negative binomial model. We also underline the inferential drawbacks related to this distribution, which limit its general use and how the Bayesian approach mitigates these problems.

The negative binomial distribution (NB) is often adopted as a two‐parameter generalization of Poisson that can take into account overdispersed count data. It also constitutes a generalization of the geometric distribution, with respect to which it allows for both overdispersion and underdispersion. Its use is well known in capture–recapture, and has also been investigated in the presence of one‐inflation in Godwin (2017).

Here we assume that the unobserved count $Y^{*}$ follows an NB model with the following parameterization in terms of r and p:

(5) $$P\left(Y^{*}=k\;|\;r,p\right)=\frac{\Gamma (k+r)}{\Gamma (r)k!}p^{r}{\left(1-p\right)}^{k},$$

and we will call the resulting model for Y one‐inflated negative binomial (OINB). In our Bayesian approach, we set two independent priors on the parameters p and r. For p we take a $Beta(\alpha_{p},\beta_{p})$ prior, while for r we compare Gamma and Inverse Gamma priors in order to evaluate the different tail behavior of these distributions on the posterior summaries.

The Gibbs sampler we developed follows the same passages presented in Section 2.1, where f(θ) takes the form (5). Recall that $n_{k}^{*}$ represents the number of units captured k times after updating $n_{0}$, Z and $Y^{*}$. Then, generating from the full conditional of p presents no difficulties, as it turns out to be:

$$\left[p\;|\;-\right]∼Beta\left(\alpha_{p}+Nr\;,\;\beta_{p}+\sum_{k>0}k\;n_{k}^{*}\right).$$

To update r, we compare two different approaches: a Gaussian random‐walk Metropolis–Hastings step and the two‐stage Gibbs sampler proposed by Zhou and Carin (2015). Note also that the presence of a Metropolis step does not preclude calculation of the marginal likelihood $p(y|M_{i})$ with Chib's approximation for the negative binomial model and for the corresponding OI counterpart, as illustrated in Chib and Jeliazkov (2001). The Appendix provides details of the marginal likelihood approximation for these models.

### 4.1 Metropolis–Hastings

The full conditional of r results in:

$$P\left(r\;|\;-\right)\propto p^{Nr}\prod_{k=0,1,\text{…}}{\left(\frac{\Gamma (k+r)}{\Gamma (r)k!}\right)}^{n_{k}^{*}}\frac{r^{\alpha_{r}-1}}{e^{r\beta_{r}}}.$$

If we consider a Gaussian random walk Metropolis–Hastings, we accept a proposed value $r^{\prime }$ with probability equal to the minimum between 1 and

$$\exp\{\sum_{k}n_{k}^{*}\left[\log\Gamma \left(r^{\prime }+k\right)-\log\Gamma \left(r^{\prime }\right)-\log\Gamma \left(r+k\right)+\log\Gamma \left(r\right)\right]+N\left(r^{\prime }-r\right)\log\left(p\right)+\Psi \},$$

where

$$\Psi =\left\{\begin{array}{cc} \left(\alpha_{r}-1\right)\log\left(r^{\prime }/r\right)+\beta_{r}\left(r-r^{\prime }\right) & \text{if}\;r∼Gamma(\alpha_{r},\beta_{r});\\ \left(\alpha_{r}-1\right)\log\left(r/r^{\prime }\right)+\beta_{r}\left(1/r-1/r^{\prime }\right) & \text{if}\;r∼InvGamma(\alpha_{r},\beta_{r}). \end{array}\right.$$

### 4.2 Two‐stage Gibbs sampler

Zhou and Carin (2015) exploit the representation of the negative binomial as a compound Poisson distribution, introduced by Quenouille (1949):

$$Y_{i}^{*}∼\text{NB}\left(r,p\right)\;⟺\;Y_{i}^{*}=\sum_{j=1}^{l_{i}}u_{i,j},$$

where

$$l_{i}∼Poisson\left(-r\log\left(p\right)\right)\;\;\text{and}\;\;u_{i,j}\overset{iid}{∼}Logarithmic\left(1-p\right).$$

They found the explicit distribution of the full conditional of $l_{i}$ to be the Chinese Restaurant Table (CRT) distribution with concentration parameter r. The two Gibbs steps are then:

(1) We sample the latent counts, $l_{i}$, associated with each observed count $y_{i}^{*}$, which can be generated as:
$$l_{i}=\sum_{j=1}^{y_{i}^{*}}v_{j},\;v_{j}∼Bernoulli\left(\frac{r}{r+j-1}\right).$$
(2) We sample r from its full conditional which, given the conjugacy between the Gamma prior for r and the Poisson distribution, results in
(6) $$\left[r\;|\;-\right]∼Gamma\left(\alpha_{r}+\sum_{i=1}^{n}l_{i}\;,\;\beta_{r}-N\log\left(p\right)\right).$$
  Note that, since the total number of captures is often in the order of thousands, and in (6) we are only interested in generating the sum of the $l_{i}$, we can simply adopt a Gaussian approximation in the first step. That is,
$$\sum_{i}l_{i}∼N\left(\sum_{i}E\left[l_{i}\right],\sum_{i}Var\left[l_{i}\right]\right).$$

### 4.3 Boundary problem

The use of the NB in capture–recapture is limited by the so called “boundary problem,” see, for example, Böhning (2015). That is, when the estimate of r approaches zero, the Horvitz–Thompson estimation of the population size diverges. More generally, when in the observed (truncated) data the mean number of captures is close to one (which is typically the case in the presence of one‐inflation), the NB model severely overestimates N, sometimes by several orders of magnitudes, even in simulated data generated by the NB itself. As pointed out in Godwin (2017), taking into account one‐inflation alleviates this phenomenon, but does not completely avoid it.

We can confirm that, even in our Bayesian approach to the OINB model, we come up against the boundary problem. In general, we noted a great sensitivity of estimates of N to small differences in the value of parameter r, particularly when r<1, and, accordingly, a great sensitivity of the estimates to specification of the prior distribution over r.

We see this phenomenon as an opportunity to investigate the usefulness of the Bayesian approach in further alleviating the boundary problem under the OINB. To this end, we conduct a simulation study to assess the effect of different prior specifications on the parameter r. We generate 100 replications of random values drawn from an OINB with parameters p=0.35, r=0.5, and ω=0.5, and we go on to test two values for N, 5000 and 500. The observed sample size n varies at each replication; its expected value over the 100 replications is 2040, and 204 when N=5000 and N=500, respectively. The values of these parameters are comparable to the values studied in Godwin (2017), in the frequentist setting, and they allow us to mimic some real cases analyzed in Section 5. All the experiments were conducted in R; the code is available as Supporting Information on the journal's web page.

We test some prior specifications on the r parameter, considering both the Gamma and the Inverse Gamma distributions. For estimation of r, we apply both the Metropolis–Hasting step and the two‐stage Gibbs sampler proposed by Zhou and Carin (2015), observing negligible differences in the results. The outcomes presented in this section are obtained using the Metropolis–Hasting approach. Finally, we compare the results with the maximum likelihood estimates for the OINB.

Table 3 shows the percentage relative bias and the percentage mean squared error (MSE) of the population size estimates, considering the difference between the true value and the mean of the posterior distribution obtained by the MCMC simulations. Table 3 also gives the number of cases, in percentage, where we encountered the boundary problem. In fact, we can define the boundary problem on both $\hat{r}$ and $\hat{N}$. We adopt the following convention: On $\hat{r}$, we set the boundary problem if $\hat{r}<0.25$, while on $\hat{N}$, this is the case if $\hat{N}>5N$. Finally, Table 3 presents the results of the maximum likelihood approach (MLE), obtained using the model proposed by Godwin (2017) and the R code provided by him as Supporting Information.

**TABLE 3:** Boundary cases for $\hat{r}$ and $\hat{N}$, %bias and %MSE of $\hat{N}$ for some prior specifications of r. Results from MLE in the bottom row, for comparison

*N* = 5000				
Prior distribution of r	% Boundary cases	% Boundary cases	% bias of $\hat{N}$	% MSE of $\hat{N}$
	for r	for N		
Gamma(0.1,0.1)	33	30	218.59	1618.82
Gamma(1,1)	11	11	97.64	859.51
InvGamma(0.1,0.1)	0	0	−10.52	6.71
InvGamma(0.5,0.5)	0	0	−15.58	5.13
InvGamma(1,1)	0	0	−19.06	5.27
InvGamma(1,2)	0	0	−26.70	7.91
MLE	16	3	91.75	2217.32

*N* = 500				
Prior distribution of r	% Boundary cases	% Boundary cases	% bias of $\hat{N}$	% MSE of $\hat{N}$
	for r	for N		
Gamma(0.1,0.1)	25	73	5043	1673356
Gamma(1,1)	0	8	249	7122
InvGamma(0.1,0.1)	0	0	−48	24
InvGamma(0.5,0.5)	0	0	−47	23
InvGamma(1,1)	0	0	−44	20
InvGamma(1,2)	0	0	−48	23
MLE	27	20	2422	584890

The Bayesian procedure implements the algorithm described in Section 4.1, setting the number of replications of the MCMC algorithm to $2\cdot 10^{6}$. We set, a priori, p(N)∝1/N, and Beta(1,1) for both ω and p. From Table 3, it can be seen that a weakly informative prior specification for r, like Gamma(1,1) can already help reduce the boundary problem, when compared to the MLE approach. The boundary problem can be yet further limited using the Inverse Gamma as prior distribution for r. In the simulation, the Inverse Gamma prior has the double advantage of reducing both the boundary problem and the MSE of the estimates, at the cost of introducing a negative bias (underestimation) of the population size N, which is more severe for small Ns. Note that we used the convention of defining the occurrence of the boundary problem when $\hat{r}<0.25$, while in Godwin (2017) the boundary problem is fixed at $\hat{r}<0.05$. We believe that $\hat{r}<0.25$ already suffices to indicate the presence of this phenomenon since, as clearly emerges from Table 3, it corresponds approximately to an estimate of N 5 times larger than its true value.

To further illustrate the performance of the NB and the OINB, with and without the boundary problem, we compare them with the models considered in Section 3 via a simulation study. In particular, we generate values from the NB with parameters N=5000, p=0.35, and from the OINB with parameters N=5000, p=0.35, and ω=0.5, under different scenarios for the size parameter r. For each scenario we generate 100 data sets and calculate the estimates of N given by the posterior mean, under the six models: Poisson, geometric, negative binomial, and their one‐inflated counterparts. Table 4 shows the average percentage relative bias and relative MSE over the 100 replicates. As we have said, the value of the parameter r appears to be crucial in identifying the boundary problem for the NB model, and, under the OINB model, ω, too, has a clear role. As a consequence, the critical values for r differ under the two models. In our data generated from the NB, with the aforementioned values for p and N, we start to observe a substantial instability in the estimates when r=0.25, and the sheer overestimation of N from the NB itself appears clearly in all simulations when r=0.1 (not showed in the table). When we generate from the OINB, estimates derived from the OINB itself start to show the same problem when r=0.5.

**TABLE 4:** Results on %bias and %MSE of $\hat{N}$

Generating model: OINB with p=0.35 and ω=0.5				
	r=0.5 (E[n]=2040)		r=1.5 (E[n]=3695)	
	% bias of $\hat{N}$	% MSE of $\hat{N}$	% bias of $\hat{N}$	% MSE of $\hat{N}$
Poi	−38.11	14.55	−7.25	0.54
Geo	5.19	0.38	42.31	17.94
NB (Gamma)	$4\cdot 10^{13}$	$9\cdot 10^{26}$	$4\cdot 10^{11}$	$2\cdot 10^{23}$
NB (InvGamma)	2518	$2\cdot 10^{5}$	$2\cdot 10^{5}$	$2\cdot 10^{10}$
OIP	−56.38	31.80	−19.32	3.74
OIG	‐29.75	8.89	12.78	1.65
OINB (Gamma)	246	2898	1.81	0.25
OINB (InvGamma)	−11.73	5.68	0.49	0.19

Generating model: NB with p=0.35				
	r=0.25 (E[n]=1154)		r=1.5 (E[n]=3965)	
	% bias of $\hat{N}$	% MSE of $\hat{N}$	% bias of $\hat{N}$	% MSE of $\hat{N}$
Poi	−71.04	50.48	−17.81	3.17
Geo	−53.99	29.17	10.98	1.21
NB (Gamma)	162.37	2044.18	0.19	0.03
NB (InvGamma)	−9.64	5.06	0.02	0.03
OIP	−74.70	55.80	−19.16	3.67
OIG	−57.52	33.11	10.91	1.20
OINB (Gamma)	5.71	64.03	−1.58	0.05
OINB (InvGamma)	−43.97	20.43	−1.86	0.06

We can see in Table 4 that, in the absence of the boundary problem, (r=1.5 in both cases), the results confirm that the two models can be safely utilized if their respective model assumptions hold; in fact, they perform better than all other competing models. As already observed in Section 3, admitting one‐inflation when it is not present leads to moderate underestimation, while ignoring one‐inflation when present causes severe overestimation of N. In fact, in all cases, the NB overestimates N by several orders of magnitude with data generated from the OINB.

A counterintuitive case is given by the data generated from the OINB with r=0.5, in which case the OINB itself results as the second best model, the best being the noninflated geometric. The explanation we gave to this result is the following: The geometric model ignores one‐inflation, and this fact should lead to an overestimation of N, but at the same time, it fixes the parameter r to 1, which is higher than the actual parameter of the generating model (r=0.5), and this fact should imply an underestimation of N. Apparently, in our simulation, these two factors balance each other, giving the geometric a better performance than the OIG and the OINB itself.

In conclusion, when the model hypothesis are met, and the boundary problem is absent or not too serious, for values of r greater than 0.25 under the NB, and greater than 0.5 under the OINB, the use of an Inverse Gamma prior may alleviate the phenomenon. However, when the problem is evident, we advise against the use of the two models.

## 5 RESULTS ON ESTIMATING ILLEGAL POPULATIONS

Illegal activities are by their very nature difficult to measure because the people involved have obvious reasons to hide them. In this section, we apply our models to estimate the number of people implicated in the exploitation of prostitution, in Italy in 2014. In addition, in Section 5.1 we illustrate the results obtained on some well‐known data sets in capture–recapture literature.

In Italy, prostitution is neither prosecuted nor regulated, but trafficking, exploitation, and aiding and abetting of prostitution are crimes subject to legal sanctions. These activities are mostly under the control of organized crime. In this study we exploit administrative records from the Ministry of Justice, which report complaints for which the judicial authority has initiated criminal proceedings.

On the basis of soft identifiers (date, country of birth, and gender), the perpetrators can be identified and followed over a given time span, which is 1 year in this application. In this way, the administrative source can be viewed as listing potential exploiters of prostitution and we can observe the number of times an individual is charged. Obviously, we cannot observe the units not captured by the Justice system. We aim to estimate the hidden part of the population, that is, the size of those unreported to the Public Prosecutor's offices. Capture–recapture models have already been used to investigate prostitution and sex workers; see, for instance, Rossmo and Routledge (1990), which estimates the number of street prostitutes in 1986/1987 in Vancouver, and Roberts Jr and Brewer (2006), which estimates the number of their clients. In this paper, we aim to estimate the size of prostitution exploiters, rather than the number of prostitutes or their clients. Our data on *prostitution exploiters* refer to perpetrators of adult sexual exploitation, according to the international classification ICCS (UNODC, 2015) ; these crimes include recruiting, enticing, or procuring a person into prostitution; pimping; keeping, managing or knowingly financing a brothel; knowingly letting or renting a building or other place for the purpose of the prostitution of others.

**FIGURE 3:**
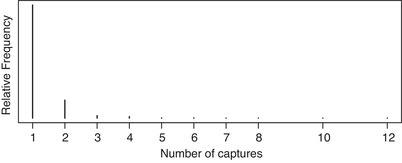
Relative frequencies of observed counts for prostitution exploitation data in Italy in 2014

Figure 3 depicts our data. The total number of observed prostitution exploiters is n=2740, the “one” counts are $n_{1}=2269$. Counts greater than 5 are relatively few; 12 is the maximum number of observed captures.

We compared all three basic models analyzed in this paper and their one‐inflated counterparts on these data. In all one‐inflated models we set a uniform ω∼Beta(1,1). We set p∼Beta(1,1) in the geometric and OIG models, and λ∼Gamma(0.01,0.01) in the Poisson and OIP. Different values for the Gamma prior were also tested, obtaining very similar results. As for the negative binomial, the boundary problem emerged clearly, as, when adopting a Gamma(0.1,0.1) prior for r, we obtained a posterior mean for N 20 times greater than any other model (498,000). For this reason, we opted for an InvGamma(0.1,0.1), both on the NB and the OINB models. In all cases, the number of replications of the MCMC algorithm is set to $10^{6}$ with a thinning of 20 observations. As priors over N, we tried both Rissanen's and the improper p(N)∝1/N. The two alternatives gave almost identical results. Standard diagnostic tools confirmed the convergence of the algorithms.

**FIGURE 4:**
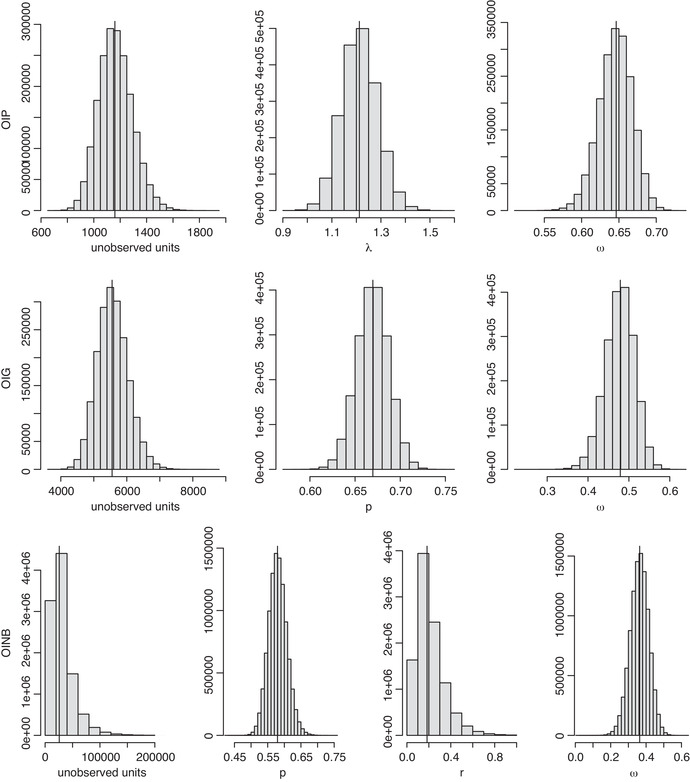
Posterior distributions of $n_{0}$ and of the parameters of all one‐inflated models for prostitution exploitation data. Vertical lines show the posterior medians

The results are summarized in Table 5 and in Figure 4. Figure 4 shows the estimated posterior distributions of $n_{0}$ and of the parameters of the one‐inflated models. The regular shape of the posterior distributions is evident from Figure 4, so the differences in adopting the posterior mode, median, or mean are quite negligible. Regularity of the posterior distributions was consistently observed in all the applications and simulations presented in this paper. Regularity of the posterior distributions does not hold for the $n_{0}$ and the r of the OINB model, due to the boundary problem.

**TABLE 5:** The posterior mode and credible intervals for the population size N, posterior mean for ω and model parameters for prostitution exploitation data

Estimator/model	$\hat{N}$	95%CI.$\hat{N}$	$\hat{\lambda }$	p	r	
Ignoring one–inflation						
Poi	7210	[6780, 7689]	0.476			
Geo	13332	[12415, 14394]			0.795	
NB	89140	[35162, 188368]		0.665	0.088	
Chao	9851	[8961, 10868]				
Zelterman	10030	[9033, 11027]	0.319			
Modeling one–inflation						$\hat{\omega }$
OIP	3895	[3656, 4156]	1.213			0.645
OIG	8182	[7406, 9233]		0.669		0.478
OINB	19566	[6174, 71710]		0.580	0.213	0.363
Mod.Chao.OIP	6493	[4163, 8823]				
Mod.Chao.OIG	19628	[9143, 30112]				

In the upper part of Table 5 we give the estimates deriving from the Poisson, geometric, and negative binomial that ignore one‐inflation and compare them to Chao and Zelterman estimators. In the lower part of the table, we give the results from the one–inflated counterparts of the 3 models and compare them to the modified Chao estimators, as suggested in Böhning et al. (2018). This estimator depends on the baseline distribution; we evaluate it assuming both Poisson and geometric distribution with one‐inflation (Mod.Chao.OIP and Mod.Chao.OIG, respectively), as in Böhning and Ogden (2021).

In Figure 3, the presence of one–inflation seems likely, and is, in fact, largely confirmed by the test introduced in Section 2.3. Both the OIP and the OIG have posterior probabilities several orders of magnitudes greater than the Poisson and the geometric. The log marginal likelihoods are: −1863.39 (Poi), −1756.23 (Geo), −1718.21 (OIG), −1761.95 (OIP). The OINB model was found to have by far the highest log marginal likelihood, namely −1712.25. However, we believe that caution should be used in adopting the estimates from the OINB. In fact, the boundary problem seems evident ($\hat{r}=0.2$), and the uncertainty contained in the estimate of $n_{0}$ is excessive (the width of the interval estimates is about 25 times greater than the total number of observed units).

As expected, if we ignore one‐inflation, we risk severely overestimating the population size. Geometric and negative binomial distributions account for heterogeneity and produce much larger estimates than the Poisson distribution.

### 5.1 Results from some popular case studies

In this section, we apply the Bayesian model to a selection of well‐known cases popular in the capture–recapture literature. We consider the following real cases:

1 Street prostitutes in Vancouver: The data show the count of prostitution arrests made by the Vancouver Police Department Vice Squad for engaging in prostitution in 1986/1987, initially presented and analyzed by Rossmo and Routledge (1990);
2 Opiate users in Rotterdam: The data show the number of applications for a methadone treatment program made by opiate users in Rotterdam in 1994, first reported and analyzed by Cruyff and van der Heijden (2008);
3 Heroin users in Bangkok: The data provide the counts of treatment episodes by heroin users in Bangkok in 2002, available in Viwatwongkasem et al. (2008) and previously analyzed by Böhning et al. (2004).

The observed count distribution of the three real cases are shown in Table 6. In the Vancouver prostitutes data set, we observe n=886 individuals and the number of units captured once is $n_{1}=541$. The Rotterdam opiate‐user data set contains n=2029 units and $n_{1}=1206$. The Bangkok heroin–user data set provides n=9302 observations with $n_{1}=2176$.

**TABLE 6:** Observed count distribution for three real cases

Real cases	Counts										
1. Prostitutes	$n_{1}$	$n_{2}$	$n_{3}$	$n_{4}$	$n_{5}$	$n_{6}$	n				
	541	169	95	37	21	23	886				
2. Opiate users	$n_{1}$	$n_{2}$	$n_{3}$	$n_{4}$	$n_{5}$	$n_{6}$	$n_{7}$	$n_{8}$	$n_{9}$	$n_{10}$	n
	1206	474	198	95	29	19	5	2	0	1	2029
3. Heroin users	$n_{1}$	$n_{2}$	$n_{3}$	$n_{4}$	$n_{5}$	$n_{6}$	$n_{7}$	$n_{8}$	$n_{9}$	$n_{10}$	$n_{11}$
	2176	1600	1278	976	748	570	455	368	281	254	188
	$n_{12}$	$n_{13}$	$n_{14}$	$n_{15}$	$n_{16}$	$n_{17}$	$n_{18}$	$n_{19}$	$n_{20}$	$n_{21}$	n
	138	99	67	44	34	17	3	3	2	1	9302

These data sets have been widely examined in capture–recapture literature, also under the one–inflation hypothesis, see Godwin and Böhning (2017) and Godwin (2017).

We apply our models to the three case–studies, with the following prior settings: For the Poisson and OIP models we set, a priori, ω∼Beta(1,1) and λ∼Gamma(0.1,0.1). In the OINB model we set r∼InvGamma(0.1,0.1) and p∼Beta(1,1). In all our applications, the number of replications of the MCMC algorithm is $10^{6}$ with a thinning of 20 observations. Standard diagnostic tools confirmed the convergence of the algorithm. The results for all three data sets are summarized in Table 7, which shows the posterior modes and credible intervals of N, and the posterior means of the model parameters.

**TABLE 7:** The posterior mode and credible intervals for the population size N, posterior mean for ω, and model parameters, for real cases

1. Prostitutes in Vancouver		$\hat{N}$	95%HPD($\hat{N})$	$\hat{\omega }$	$\hat{\lambda }$	$\hat{r}$	$\hat{p}$
Model	Poi	1240	1177–1300		1.254		
	Geo	2045	1906–2217				0.570
	NB	3340	1977–167925			0.145	0.395
	OIP	1017	982–1058	0.438	2.037		
	OIG	1820	1669–2003	0.192			0.517
	OINB	1040	991–1238	0.399		19.104	0.862
	Mod.Chao.OIP	1005	933–1077				
	Mod.Chao.OIG	1421	1097–1745				

2. Opiate users in Rotterdam		$\hat{N}$	95%HPD($\hat{N})$	$\hat{\omega }$	$\hat{\lambda }$	$\hat{r}$	$\hat{p}$
Model	Poi	2934	2832–3038		1.174		
	Geo	4913	4676–5188				0.588
	NB	4960	4244–6818			0.869	0.566
	OIP	2500	2418–2587	0.336	1.663		
	OIG	4796	4491–5085	0.047			0.577
	OINB	3213	2616–4665	0.157		2.861	0.692
	Mod.Chao.OIP	2633	2398–2867				
	Mod.Chao.OIG	4745	3691–5799				

3. Heroin users in Bangkok		$\hat{N}$	95%HPD($\hat{N})$	$\hat{\omega }$	$\hat{\lambda }$	$\hat{r}$	$\hat{p}$
Model	Poi	9452	9427–9477		4.134		
	Geo	12206	12064–12341				0.238
	NB	11572	11357–11817			1.232	0.267
	OIP	9364	9349–9380	0.207	5.004		
	OIG	12195	12056–12334	0.003			0.237
	OINB	10826	10606–11098	0.056		1.627	0.302
	Mod.Chao.OIP	9859	9757–9961				
	Mod.Chao.OIG	11810	11350–12270				

The presence of one‐inflation in these data sets is less severe than in the prostitution exploitation data analyzed in the previous section. However, as expected, estimates from the base distributions are consistently greater than the corresponding one‐inflated estimates, confirming that we might be overestimating the population size if we ignore one‐inflation.

For the Vancouver prostitute data, our model selection strategy strongly suggests the OINB distribution, its posterior probability being several orders of magnitudes greater than the competing models. The inflation rate ω is estimated around 0.40. The base negative binomial encounters the boundary problem, as is clear from the r estimate and even more from the credible intervals for N. OINB and OIP models produce similar estimates for N, with the credible intervals mostly overlapping (the 95%HPD under OINB is slightly greater than under OIP), while the OIG's credible interval barely overlaps the others.

As for the Rotterdam opiate‐user data, Bayesian model selection largely favors the geometric distribution, with a posterior probability of 0.89, against 0.104 and 0.006 for OIG and OINB, respectively; the Poisson models posterior probabilities being negligible, both the baseline and the one‐inflated. In this case, the one‐inflation does not seem to affect the data.

The posterior model probabilities for Bangkok heroin‐user data favor the OINB model, even though the estimated inflation rate is quite low, a mere 0.056. The boundary problem is not an issue with this data set, since the estimate of r is rather greater than 1.

In all cases, the OINB model produces estimates for N higher than the OIP and lower than OIG. Also the one‐inflation rate estimates under the OINB model prove always lower than the estimates obtained from the OIP model and higher than those from the OIG. It appears that by using the OINB, part of the one‐inflation component identified by the OIP is instead explained through the two parameters of the negative binomial. The credible intervals of the OIP are consistently smaller than those of the competing models, and barely overlap, with the exception of Vancouver prostitute data, where actually the OINB model tends to the OIP one (note the high estimates for the parameter r).

The results in Table 7 can be compared with non Bayesian results reported in Godwin and Böhning (2017) and Godwin (2017), for the OIP and negative binomial models. We note that the use of weakly informative priors leads to results that are close to the frequentist approach. Moreover, the results from our Bayesian model selection strategy are also confirmed by likelihood ratio tests proposed in Godwin (2017), even if likelihood ratio tests provide less strong evidence than our results.

## 6 CONCLUDING REMARKS AND FUTURE WORKS

In this paper we have dealt with the issue of one‐inflation on repeated count data in population size estimation, adopting a fully Bayesian approach. We discussed our model for one‐inflation under an unspecified count distribution, describing a general Gibbs sampler. Specifically, we derived the conditional distributions of the model parameters under the Poisson and geometric assumption; moreover, to deal with data that show overdispersion, we also illustrated the Bayesian analysis for the negative binomial model. We considered the boundary problem of the negative binomial distribution; in the Bayesian setting the prior parameter specification might help alleviate it. A fully Bayesian model selection approach, which includes testing for the one‐inflation assumption, was developed for all the distributions considered in the paper.

Alongside the usual advantages of a Bayesian approach, namely, the possibility of incorporating any prior knowledge in the analysis and ease in producing interval estimates of any quantity as a by‐product of the estimation procedure, we recognize a less obvious point in favor. In fact, although, admittedly, it is not common to have prior information on the quantities at hand, even weakly informative priors can have a positive impact on the analysis. As we saw in Section 4.3, the use of a weakly informative prior when using a negative binomial model or its one‐inflated counterpart can help stabilize the estimation procedure and avoid the “boundary problem” in case of moderate severity. On the other hand, the choice of the prior distribution for the size parameter of the negative binomial may affect model selection procedures, which require additional investigation in order to allow a more general use of such distribution in capture–recapture models.

We are currently working on extensions of the current model to cope with observed and unobserved heterogeneity in the presence of one‐inflation, exploiting individual covariates, and introducing more complex hierarchical structures and mixing models.

Moreover, we are considering the possibility of taking model uncertainty into account with a model averaging technique in a single procedure by exploiting the reversible jump algorithm (see Green, 1995).

In addition, when dealing with sensible data, like the prostitution exploitation data, which do not share a unique identifier, we may encounter record linkage problems. In this case, it would be important also to take into account the record linkage process uncertainty in population size estimation; see Tancredi and Liseo (2011). Note also that linkage errors can themselves produce one‐inflation. In fact, when matching information does not suffice to recognize multiple captures of the same individual, the resulting missing links erroneously increase the number of singletons. However, it is worth nothing that, unlike the case with the framework considered in this paper, linkage errors also affect the observed sample size n.

Finally, we are investigating more general behavioral mechanisms producing different forms of inflation. For example, we could assume that when the latent count $y^{*}$ is equal to k, instead of necessarily having an observation y equal to 1 or to the true value k, we have that y follows a mixture of two distributions. In particular we may have a mixture component with weight 1−ω concentrated on the latent value $y^{*}=k$. The other component with weight ω may have support on the set {1,…,k} and can, for example, be a Binomial(k,ψ) truncated on 0. Thus, when ψ=0 we have exactly the form of inflation discussed in this paper while when ψ>0 the model also allows us to inflate counts greater than one, generalizing the effects of the behavioral mechanism.

## CONFLICT OF INTEREST

The authors have declared no conflict of interest.

### OPEN RESEARCH BADGES

This article has earned an Open Data badge for making publicly available the digitally‐shareable data necessary to reproduce the reported results. The data is available in the Supporting Information section.

This article has earned an open data badge “**Reproducible Research**” for making publicly available the code necessary to reproduce the reported results. The results reported in this article could fully be reproduced.

## Supporting information

Supporting Information

## Data Availability

Data sharing not applicable – no new data generated.

## MARGINAL LIKELIHOOD CALCULATIONS

Expression (4) for the marginal likelihood is obtained by observing that

p(y|Mi)=∫∑N=n∞f(y|θi,N,Mi)p(N)p(θi)dθi=∫∑N=n∞Nnf(0|θi)N−n∏i=1nf(yi|θi)cNp(θi)dθi=c∫∑n0=0∞(n+n0)!n!n0!1n+n0f(0|θ)n0(1−f(0|θi))n∏i=1nf(yi|θi)1−f(0|θi)p(θi)dθi=cn∫∑n0=0∞n+n0−1n−1f(0|θ)n0(1−f(0|θi))n∏i=1nf(yi|θi)1−f(0|θi)p(θi)dθi=cn∫∏i=1nf(yi|θi)1−f(0|θi)p(θi)dθi.

Chib's approximation is based on the identity

$$p\left(y|M_{i}\right)=\frac{f\left(y|\theta_{i},N\right)p\left(\theta_{i}\right)p\left(N\right)}{p(\theta_{i},N|y,M_{i})}$$

valid for each point $\left(\theta_{i},N\right)$. To approximate the marginal likelihood we may select a point $({\tilde{\theta }}_{i},\tilde{N}$) given, for example, by the posterior means obtained with a first run of the Gibbs sampler and then estimate the value of the posterior $p({\tilde{\theta }}_{i},\tilde{N}|y,M_{i})$ via a second run by using the following strategies.

For the Poisson model $M_{i}$, where $\theta_{i}=\lambda$, suppressing the model dependence in the notation hereafter, we have $p\left(\tilde{\theta },\tilde{N}|y\right)=p\left(\tilde{\lambda },\tilde{N}|y\right)=p\left(\tilde{N}|\tilde{\lambda },y\right)p\left(\tilde{\lambda }|y\right)$ and the only quantity that needs to be estimated is $p(\tilde{\lambda }|y)$. Anyway

$$p\left(\tilde{\lambda }|y\right)=\sum_{N}p\left(\tilde{\lambda },N|y\right)=\sum_{N}p\left(\tilde{\lambda }|y,N\right)p\left(N|y\right),$$

and by exploiting the T realizations $N_{\left(1\right)}\text{…},N_{\left(T\right)}$ of p(N|y) from a second run of the Gibbs sampler we can estimate $p(\tilde{\lambda }|y)$ by

$$p\left(\tilde{\lambda }|y\right)\approx \frac{1}{T}\sum_{t=1}^{T}p\left(\tilde{\lambda }|y,N_{\left(t\right)}\right),$$

where $p(\tilde{\lambda }|y,N_{\left(t\right)})$ is the density of a Gamma$\left(\alpha_{\lambda }+s,\beta_{\lambda }+N_{\left(t\right)}\right)$.

Similarly, for the geometric model, where θ=p, we have $p\left({\tilde{\theta }}_{i},\tilde{N}|y\right)=p\left(\tilde{p},\tilde{N}|y\right)=p\left(\tilde{N}|\tilde{p},y\right)p\left(\tilde{p}|y\right)$ and the only quantity that needs to be estimated is $p(\tilde{p}|y)$. Anyway

$$p\left(\tilde{p}|y\right)=\sum_{N}p\left(\tilde{p},N|y\right)=\sum_{N}p\left(\tilde{p}|y,N\right)p\left(N|y\right)$$

and by exploiting the T realizations $N_{\left(1\right)}\text{…},N_{\left(T\right)}$ of p(N|y) from a second run of the Gibbs sampler we can estimate $p(\tilde{p}|y)$ by

$$p\left(\tilde{p}|y\right)\approx \frac{1}{T}\sum_{t=1}^{T}p\left(\tilde{p}|y,N_{\left(t\right)}\right),$$

where $p(\tilde{p}|y,N_{\left(t\right)})$is the density of a Beta $\left(\alpha_{p}+N_{\left(t\right)},\beta_{p}+s\right)$.

For the OIP model where θ=(λ,ω) we have $p\left(\tilde{\theta },\tilde{N}|y\right)=p\left(\tilde{\lambda },\tilde{\omega },\tilde{N}|y\right)=p\left(\tilde{N}|\tilde{\lambda },\tilde{\omega },y\right)p\left(\tilde{\lambda },\tilde{\omega }|y\right)$. In this case we need to estimate $p(\tilde{\lambda },\tilde{\omega }|y)$ where

$$p\left(\tilde{\lambda },\tilde{\omega }|y\right)=\sum_{N}\sum_{y^{*}}p\left(\tilde{\lambda },\tilde{\omega },N,y^{*}|y\right)=\sum_{N}\sum_{y^{*}}p\left(\tilde{\lambda },\tilde{\omega }|y,N,y^{*}\right)p\left(N,y^{*}|y\right).$$

Then, by exploiting the T realizations $y_{\left(1\right)}^{*},N_{\left(1\right)},\text{…},y_{\left(T\right)}^{*},N_{\left(T\right)}$ of $p(y^{*},N|y)$ from the the first Gibbs sampler run, we can estimate $p(\tilde{\lambda },\tilde{\omega }|y)$ by

$$p\left(\tilde{\lambda },\tilde{\omega }|y\right)\approx \frac{1}{T}\sum_{t=1}^{T}p\left(\tilde{\lambda },\tilde{\omega }|y,y_{\left(t\right)}^{*},N_{\left(t\right)}\right).$$

Note that λ and ω are conditionally independent given $y,y^{*}$ and N. Moreover the conditional distribution $\lambda |y,y^{*},N$ is Gamma$\left(\alpha_{l}+\sum_{k>0}kn_{k}^{*},\beta_{l}+N\right)$ while the conditional distribution $\omega |y,y^{*},N$ is Beta$\left(\alpha_{\omega }+n_{z},\beta_{\omega }+\sum_{k>1}n_{k}\right)$.

Similarly, for the OIG model where θ=(p,ω) we can follow exactly the same strategy by factorizing the posterior distribution as $p\left(\tilde{\theta },\tilde{N}|y\right)=p\left(\tilde{p},\tilde{\omega },\tilde{N}|y\right)=p\left(\tilde{N}|\tilde{p},\tilde{\omega },y\right)p\left(\tilde{p},\tilde{\omega }|y\right)$ and estimating $p(\tilde{p},\tilde{\omega }|y)$ by

$$p\left(\tilde{p},\tilde{\omega }|y\right)\approx \frac{1}{T}\sum_{t=1}^{T}p\left(\tilde{p},\tilde{\omega }|y,y_{\left(t\right)}^{*},N_{\left(t\right)}\right).$$

where $y_{\left(1\right)}^{*},N_{\left(1\right)},\text{…},y_{\left(T\right)}^{*},N_{\left(T\right)}$ are T realizations from $p(y^{*},N|y)$ obtained from the first Gibbs sampler run. Also in this case $\tilde{p}$ and $\tilde{\omega }$ are conditionally independent given $y,y^{*},N$. The conditional distribution $p|y,y^{*},N$ is Beta($\alpha_{p}+N,\beta_{p}+\sum_{k>0}n_{k}^{*}$) while and $\omega |y,y^{*},N$ is Beta$\left(\alpha_{\omega }+n_{z},\beta_{\omega }+\sum_{k>1}n_{k}\right)$.

For the negative binomial model we have θ=(p,r) and the posterior can be factorized as

$$\begin{array}{ccc} p(\tilde{\theta },\tilde{N}|y) & = & p\left(\tilde{p},\tilde{r},\tilde{N}|y\right)=p\left(\tilde{N}|\tilde{p},\tilde{r},y\right)p\left(\tilde{p}|\tilde{r},y\right)p\left(\tilde{r}|y\right), \end{array}$$

where, as in the previous models, the conditional density $p(\tilde{N}|\tilde{p},\tilde{r},y)$ is known. The conditional density $p(\tilde{p}|\tilde{r},y)$ can be obtained by an extra run of the Gibbs sampler with r fixed to $\tilde{r}$. In fact

$$p\left(\tilde{p}|\tilde{r},y\right)=\sum_{N}p\left(\tilde{p},N|\tilde{r},y\right)=\sum_{N}p\left(\tilde{p}|N,\tilde{r},y\right)p\left(N|\tilde{r},y\right)$$

and the conditional distribution $p|N,\tilde{r},y$ is Beta with parameters $\alpha_{p}+Nr,\beta_{p}+s$. Instead the calculation of the marginal posterior $p(\tilde{r}|y)$ can be obtained following the approach proposed by Chib and Jeliazkov (2001).

For the OI negative binomial we have θ=(p,r,ω) and the posterior can be factorized as

$$\begin{array}{ccc} p(\tilde{\theta },\tilde{N}|y) & = & p\left(\tilde{p},\tilde{\omega },\tilde{r},\tilde{N}|y\right)=p\left(\tilde{N}|\tilde{p},\tilde{r},\tilde{\omega },y\right)p\left(\tilde{\omega },\tilde{p}|\tilde{r},y\right)p\left(\tilde{r}|y\right). \end{array}$$

Also in this case the conditional density $p(\tilde{N}|\tilde{\omega },\tilde{p},\tilde{r},y)$ is known and $p(\tilde{\omega },\tilde{p}|\tilde{r},y)$ can be obtained by an extra run of the Gibbs sampler with r fixed to $\tilde{r}$ by

$$p\left(\tilde{p},\tilde{\omega }|\tilde{r},y\right)\approx \frac{1}{T}\sum_{t=1}^{T}p\left(\tilde{p},\tilde{\omega }|\tilde{r},y,y_{\left(t\right)}^{*},N_{\left(t\right)}\right).$$

Note that the parameters p and ω are conditionally independent given $r,y,y^{*}$, and N with $p|r,y,y^{*}$, and N, which is Beta$\left(\alpha_{p}+Nr,\beta_{p}+\sum_{k>0}kn_{k}^{*}\right)$ and $\omega |y,y^{*},N$ which is, as in the previous inflated models, Beta$\left(\alpha_{\omega }+n_{z},\beta_{\omega }+\sum_{k>1}n_{k}\right)$. Finally, as for the noninflated negative binomial counterpart, the calculation of the marginal posterior $p(\tilde{r}|y)$ can be obtained following the approach proposed by Chib and Jeliazkov (2001).
